# Callus, Dedifferentiation, Totipotency, Somatic Embryogenesis: What These Terms Mean in the Era of Molecular Plant Biology?

**DOI:** 10.3389/fpls.2019.00536

**Published:** 2019-04-26

**Authors:** Attila Fehér

**Affiliations:** ^1^Department of Plant Biology, University of Szeged, Szeged, Hungary; ^2^Institute of Plant Biology, Biological Research Centre, Hungarian Academy of Sciences, Szeged, Hungary

**Keywords:** callus, dedifferentiation, plant regeneration, plant cell and tissue culture, somatic embryogenesis, terminology, totipotency

## Abstract

Recent findings call for the critical overview of some incorrectly used plant cell and tissue culture terminology such as dedifferentiation, callus, totipotency, and somatic embryogenesis. Plant cell and tissue culture methods are efficient means to preserve and propagate genotypes with superior germplasm as well as to increase genetic variability for breading. Besides, they are useful research tools and objects of plant developmental biology. The history of plant cell and tissue culture dates back to more than a century. Its basic methodology and terminology were formulated preceding modern plant biology. Recent progress in molecular and cell biology techniques allowed unprecedented insights into the underlying processes of plant cell/tissue culture and regeneration. The main aim of this review is to provide a theoretical framework supported by recent experimental findings to reconsider certain historical, even dogmatic, statements widely used by plant scientists and teachers such as “plant cells are totipotent” or “callus is a mass of dedifferentiated cells,” or “somatic embryos have a single cell origin.” These statements are based on a confused terminology. Clarification of it might help to avoid further misunderstanding and to overcome potential “terminology-raised” barriers in plant research.

## Introduction – a Short Historical Preview

Plants exhibit a remarkable developmental plasticity. This is manifested, among others, in their high regeneration capacity. Plants, from time to time, need to cope with physical damages caused by their biotic or abiotic environment. To ensure survival, they have dedicated developmental pathways to close injuries and/or replace lost parts/organs. These pathways have been exploited for vegetative plant propagation long since. Besides, the regeneration ability of plants attracted scientific interest as early as the end of the 19th century (for a recent review of plant cell culture history see Sugiyama, 2015). Histological wound responses and callus formation had been observed and the term “dedifferentiation” was already used at this early period. The start of *in vitro* plant cell and tissue culture research is dated to 1902, when Gottlieb Haberlandt presented his hypothesis on the intrinsic capability of isolated plant cells for autonomous life (Haberlandt, 1902). Long-term proliferation and maintenance of cultured plant tissues were worked out during the 1930th and provided experimental proofs for this hypothesis. It was followed by the observation that the phytohormones auxin and cytokinin are both required for *in vitro* cell proliferation. Moreover, it was revealed that the ratio of these hormones determines the morphogenetic pathway that the *in vitro* cultured tissue will follow: high and low ratios of cytokinin to auxin favored shoot and root regeneration, respectively, whereas more balanced concentrations resulted in unorganized growth of a cell mass (Skoog and Miller, 1957). This proliferating cell mass was termed as “callus” due to its resemblance to the wound-healing plant tissue. In the late 1950th, it was proved that besides sequential shoot and root organogenesis whole plants can be regenerated from cultured plant cells in only one step via embryo formation (Steward et al., 1958; Reinert, 1959). This pathway was later termed as “somatic embryogenesis” and its initiation was confined to single cells (Backs-Hüsemann and Reinert, 1970). This process was considered to be the experimental proof of the “totipotency” of plant cells, namely that each somatic plant cell has the capability to regenerate into an entire plant. This view was further supported by the isolation and culture of leaf protoplasts (single cells devoid of cell wall) and their development into whole plants (Takebe et al., 1971). Based on the above studies, plant cell/tissue culture and regeneration systems were successfully applied for plant propagation in the case of hundreds of plant species and their various explants. Therefore, the view formulated by Steward and colleagues in 1970 that “in principle, all normally diploid somatic cells are essentially totipotent and that present failures to rear them into plants merely present the challenge to find the right conditions for their development” (Steward et al., 1970) became widely accepted. It was also commonly believed that dedifferentiation of somatic plant cells is a prerequisite of subsequent plant regeneration. Recent research, however, has resulted in deeper insights into the above processes and questioned several of the above historical, sometimes even dogmatic, statements of plant cell and tissue culture. Some of the most critical issues are briefly discussed below.

## Dedifferentiation and Callus Formation

**FIGURE 1:**
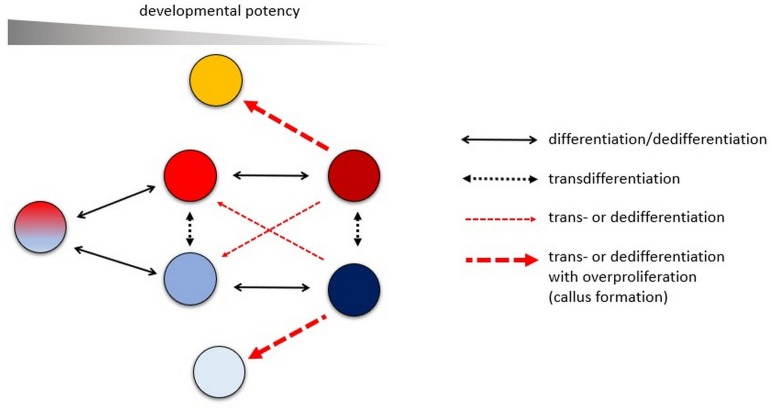
The various differentiation pathways a plant cell can follow and the used terminology to describe them. Differentiation is generally associated with decreased, dedifferentiation with increased developmental potency. In a strict sense, dedifferentiation can take place only within the same developmental lineage and can be considered as the reversion of differentiation. Transdifferentiation is used to describe cell fate changes independent of developmental potency. However, in plant biology, transdifferentiation leading to increased developmental potency is often referred to as dedifferentiation, especially during callus formation. Callus formation is not a step back in the developmental lineage but rather the result of overproliferation/transdifferentiation of differentiated cells. Some or most of the cells of the heterogenous callus tissue can have increased developmental potency.

The term “dedifferentiation” has many definitions: “process by which mature or specialized cells lose their differentiated character and rejuvenate” (Bloch, 1941); “a process in which tissues that have undergone cell differentiation can be made to reverse the process so as to become a primordial cell again” (Hale et al., 2005); “involves a terminally differentiated cell reverting back to a less differentiated stage from within its own lineage” (Jopling et al., 2011); “its distinguishing feature is the withdrawal from a given differentiated state into a ‘stem cell’-like state that confers pluripotentiality” (Grafi, 2004). The common in these definitions is that, contrary to differentiation, dedifferentiation increases the developmental potency of cells. There is a controversy, however, to what extent the term “dedifferentiation” can be used. Is it the reversion of differentiation and therefore can take place only within the same cell lineage (Hale et al., 2005; Jopling et al., 2011; Sugimoto et al., 2011) or can be used for all processes increasing cellular potency (e.g., Grafi, 2004; Figure 1)? Crossing the barriers between cell lineages is generally considered as transdifferentiation irrespectively of the developmental potency of the cells (Sugimoto et al., 2011; Figure 1).

One of the main cause of controversies is the mixing of the genetic and developmental biology viewpoints of cellular differentiation. All multicellular organism is characterized by a given number of genes, but none of their cells express all but only a portion of them and as such can be considered as genetically differentiated. In consequence, a genetically fully dedifferentiated cell would express all the genes coded in the genome. Such cell obviously does not exist. Even the zygote having the highest developmental potency have a well-defined gene expression pattern (Sprunck et al., 2005; Zhao et al., 2011; Abiko et al., 2013; Domoki et al., 2013; Leljak-Levanić et al., 2013) and from a genetic point of view is differentiated to fulfill its specific function, the initiation of the autonomous development of the organism.

From a developmental biology perspective, the zygote is the origo of the cell differentiation process. Therefore, it is often considered to be the “least-differentiated” cell of the organism, or “dedifferentiated.” Similarly, stem cells are also considered as “less differentiated” than somatic cells or “dedifferentiated.” However, neither of these cells were formed by dedifferentiation (i.e., via the reversion of differentiation or loosing differentiated functions) and therefore the use of the term “dedifferentiated” in this context is irrelevant. Despite their high developmental potencies, stem cells are also differentiated: the specific cells of the shoot or root meristems have well defined gene expression patterns depending on meristem identity factors (e.g., Birnbaum et al., 2003; Yadav et al., 2009) and the pluripotent embryonic stem cells of animals express the four yamanaka factors regulating several stem cell specific genes (e.g., Kulcenty et al., 2015). Stem cells have the function to sense and respond to stem cell niche signals, express cell fate determinants, segregate those into specific cellular regions and then divide asymmetrically to ensure self-renewal and the production of progenitor cells. These are specific functions that require the action of a specific set of genes, what was ensured by cell differentiation yet allowing a high developmental potency. The term “dedifferentiated” is erroneously used to indicate the developmental potency of these cells. In my view, the terms “zygote,” “stem cell,” “cancer cell,” “callus cell,” “somatic cell” well describe the various differentiated cell states without additional attributes. The qualifiers “differentiated,” “dedifferentiated,” “transdifferentiated” should only be used to indicate the way the given cell was formed, but not the end state.

Dedifferentiation, similarly, to differentiation, is a transient process that governs cells from one differentiated state to another. A cell can only be regarded as differentiated or dedifferentiated in relation to another one, namely to the one it derived from. General “differentiated” or “dedifferentiated” cell states do not exist. As differentiation results in various specialized cell types, dedifferentiation, the opposite process, does the same. One important difference is that during dedifferentiation the cell’s developmental potency increases. Crossing the barriers between cell lineages is generally considered as transdifferentiation irrespectively of the developmental potency of the cells (Sugimoto et al., 2011; Figure 1). In the terminology of plant cell and tissue culture, however, dedifferentiation is collectively used for all processes resulting in increased developmental potencies (Figure 1). It is not surprising, if we consider that cell lineages are less important in plant than in animal development and plant somatic cells can be more easily reprogrammed (Gaillochet and Lohmann, 2015).

In plants, “dedifferentiation” is strongly associated with callus formation since callus is widely regarded as a proliferating mass of “dedifferentiated cells.” However, as it was outlined above, dedifferentiation in a strict sense is the reversion of differentiation, but callus formation is not, since the differentiated cell was not formed from a callus (Figure 1). Callus formation can rather be considered as a type of transdifferentiation (Sugimoto et al., 2011). Furthermore, as it was also discussed above, a general “dedifferentiated cell state” does not exist.

Recent transcriptomic data support the view that calli can be formed via various initial pathways which converge on the same gene regulatory network coordinating stress, hormone, and developmental responses. Nevertheless, the gene sets expressed in various types of calli only partly overlap.

Auxin-induced (incubation on callus-induction medium, CIM; Valvekens et al., 1992) callus formation on *in vitro*-cultured Arabidopsis explants follows the lateral root development pathway (Atta et al., 2009; Sugimoto et al., 2010). This type of callus was shown to express root meristem (pluripotency) markers in a more-or-less correct temporal and spatial organization (Sugimoto et al., 2010). Similarly, to lateral root primordia (LRPs), auxin-induced callus formation initiates in pericycle cell-like stem cells and there is no requirement for preceding dedifferentiation of differentiated somatic cells (Atta et al., 2009; Sugimoto et al., 2010, 2011). This callus type at the early developmental phase might be considered as an over proliferating lateral root primordium. Most remarkably, these characteristics of the calli were independent of the type of the explant (either root or aerial organs) excluding also the possibility of dedifferentiation in a strict sense (within cell lineage).

**Table 1:** Common up-regulated genes in various auxin-induced callus tissues (see Figure 2B for details).

Gene ID	Symbol	Description
AT1G02850	BGLU11	Beta glucosidase 11
AT1G19850	MP	Transcriptional factor B3 family protein/auxin-responsive factor
AT1G33790	AT1G33790	Jacalin lectin family protein
AT1G55610	BRL1	BRI1 like
AT2G32280	AT2G32280	GPI inositol-deacylase C, putative (DUF1218)
AT2G39350	ABCG1	ABC-2 type transporter family protein
AT2G43510	TI1	Trypsin inhibitor protein 1
AT2G47260	WRKY23	WRKY DNA-binding protein 23
AT3G01970	WRKY45	WRKY DNA-binding protein 45
AT3G02210	COBL1	COBRA-like protein 1 precursor
AT3G13380	BRL3	BRI1-like 3
AT3G14060	AT3G14060	Hypothetical protein
AT3G15720	AT3G15720	Pectin lyase-like superfamily protein
AT3G25730	EDF3	Ethylene response DNA binding factor 3
AT3G29810	COBL2	COBRA-like protein 2 precursor
AT3G48410	AT3G48410	Alpha/beta-hydrolases superfamily protein
AT3G48580	XTH11	Xyloglucan endotransglucosylase/hydrolase 11
AT3G62860	AT3G62860	Alpha/beta-hydrolases superfamily protein
AT4G02280	SUS3	Sucrose synthase 3
AT4G15910	DI21	Drought-induced 21
AT4G27260	WES1	Auxin-responsive GH3 family protein
AT4G36930	SPT	Basic helix-loop-helix (bHLH) DNA-binding superfamily protein
AT4G37870	PCK1	Phosphoenolpyruvate carboxykinase 1
AT4G38210	EXPA20	Expansin A20
AT4G38580	FP6	Farnesylated protein 6
AT5G10510	AIL6	AINTEGUMENTA-like 6
AT5G14000	NAC084	NAC domain containing protein 84
AT5G17980	AT5G17980	C2 calcium/lipid-binding plant phosphoribosyltransferase family protein
AT5G26220	AT5G26220	ChaC-like family protein
AT5G49690	AT5G49690	UDP-Glycosyltransferase superfamily protein
AT5G50260	CEP1	Cysteine proteinases superfamily protein

**FIGURE 2:**
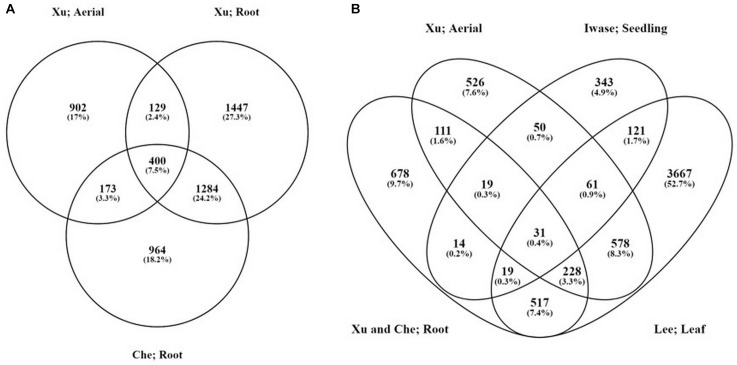
The overlaps among various gene expression data sets obtained form analyses of auxin-induced calli. **(A)** Comparison of up-regulated genes in root and aerial explants (hypocotyl and cotyledon) at the time of initial callus formation (at 4 days on callus-induction medium) in relation to the initial explant (the data were obtained from the experiments of Xu et al., 2012 and Che et al., 2006). **(B)** Comparison of up-regulated genes in root (only the genes that were found to be up-regulated by both Xu et al., 2012 and Che et al., 2006 were used) and aerial explants (hypocotyl and cotyledon; Xu et al., 2012) at the time of initial callus formation and in established calli induced on seedlings (Iwase et al., 2011a) or leaves (Lee et al., 2016) in the presence of auxin. The diagrams were made by the “Venny” online tool (Oliveros, 2007/2015).

In two independent experiments, 5488 and 4939 genes were found to be regulated, respectively, during auxin-induced callus formation from root explants after the fourth day of culture (Che et al., 2006; Xu et al., 2012). Only 2656 of the genes showed an overlap in the two studies. Considering callus formation from different explants, seedling roots were compared to aerial parts (hypocotyls and cotyledons) by Xu et al. (2012). There were 529 upregulated genes that were present in both datasets, while 1075 gene was upregulated only in the aerial tissue-derived calli and 2731 in the root-derived ones (Xu et al., 2012; Figure 2A). Comparison of these data sets to those obtained from auxin-induced leaf- (Lee et al., 2016) and seedling-derived (Iwase et al., 2011a) established callus tissues, only 31 common transcripts could be identified (Figure 2B and Table 1). The above numbers indicate that the type of explant and the other experimental conditions have considerable effects on the number and specificity of genes that are regulated during auxin-induced callus development.

In addition to auxin, callus may also form in response to other hormones or wounding (for review, Ikeuchi et al., 2013). Wound-induced calli do not express LRP markers and their formation is regulated by the transcription factors WOUND-INDUCED DEDIFFERENTIATION (WIND) 1–4 (Iwase et al., 2011a,b, 2015; Ikeuchi et al., 2013, 2016, 2017). Wounding up-regulates cytokinin biosynthesis and signaling, leading to the activation of cell proliferation and callus formation (Iwase et al., 2011a,b; Ikeuchi et al., 2017). Interestingly, endogenous auxin accumulation or activation of auxin response could not be detected at the wound site and the auxin signaling mutant *solitary root* had no defects in wound-induced callus formation (Iwase et al., 2011a,b; Ikeuchi et al., 2017). Therefore, exogenous auxin and wounding triggers callus formation in different ways. Iwase et al. (2011a) compared the gene expression of calli formed on Arabidopsis seedlings due to ectopic WIND1 expression, or 2,4-D-treatment, respectively. There was a significant gene expression overlap among the WIND1- and auxin-induced calli (326 genes) while 735 genes were upregulated only in response to WIND1 but not for 2,4-D and 641 genes were regulated in the opposite way (Iwase et al., 2011a). All the 31 up-regulated genes common in auxin-induced calli (Figure 2B and Table 1) are also upregulated by WIND1. This rather limited gene set including many transcription factors might be related to specialized callus traits/functions.

The above comparison of gene expression data based on a few time points and inducing agents allows only limited conclusions about the genetic nature of callus tissues in general. Recently, Ikeuchi and co-workers followed a more straightforward approach to delineate a gene regulatory network underlying callus formation. They established regulatory relationships among 252 transcription factors and 48 promoters using a systematic yeast one-hybrid screening approach. It was found that the auxin- and wound-induced callus formation pathways converge on the same gene regulation network, the core elements of which are the PLT3, ESR1, and HSFB1 transcription factors (Ikeuchi et al., 2018). This study also highlights that specialized callus functions including developmental potencies rely on the cooperative action of defined sets of transcription factors and not merely on the loss of differentiated functions.

Gain- or loss-of-function of many cell cycle or developmental regulators might also result in callus formation (Ikeuchi et al., 2013). Whether these pathways overriding cell differentiation also converge on the above gene regulatory node is an interesting question to be investigated. Furthermore, it also needs to be investigated how genetically homogeneous a callus tissue is? Is it a mass of more-or-less uniform dividing cells with similar developmental potencies or have cells with various potencies/fates/functions similarly to the blastema tissue of animals (Birnbaum and Alvarado, 2008)? Callus seems to be rather heterogenous during its formation (e.g., calli formed from lateral root primordia expressing root meristem markers in a partially regulated way Sugimoto et al., 2011). Only certain cells of calli but not all of them can be involved in organ regeneration or embryogenesis, supporting a heterogenous organization. It must be emphasized here that developmental potency is a cellular term and, therefore, a callus cannot be pluri- or totipotent but can have pluri- or totipotent cells (see also further). Long-term callus cultures become more and more homogenous often with parallel loss of developmental potencies, especially in liquid culture (cell suspension cultures).

In addition to callus formation, protoplast isolation is also strongly believed to be associated with plant cell dedifferentiation (Zhao et al., 2001; Williams et al., 2003; Grafi, 2004; Chupeau et al., 2013). During protoplast isolation, the tissues are wounded, the cells are exposed to cell wall-digesting enzymes, separated from each other, and released into an artificial medium. As a result, the stressed cells lose their developmental and hormonal constrains and differentiated functions (Williams et al., 2003; Avivi et al., 2004; Koukalova et al., 2005; Chupeau et al., 2013). These events as well as the associated gene expression changes are rather similar to those characterizing cellular senescence (Damri et al., 2009). In agreement, these protoplasts die in a hormone-free medium. It is hypothesized that senescing leaf cells go through dedifferentiation similarly to isolated protoplasts (Damri et al., 2009), however, rather the isolated protoplasts go through senesce similarly to the cells in a senescing leaf. Although senescence is characterized by the progressive loss of differentiated cellular functions, it is considered to represent a special case of transdifferentiation/metaplasia and not dedifferentiation (Thomas et al., 2003).

Leaf senescence can be reverted until the final degradation state such as protoplasts can be kept alive in the presence of cytokinin and/or auxin. However, in the absence of proper developmental signals, protoplast-derived cells cannot be reverted to mesophyll cells; they develop to elongated or proliferating parenchymatic (callus) cells in the presence of auxin or auxin and cytokinin, respectively (Grafi, 2004). The continuous presence of the two hormones finally leads to the formation of callus tissue. The formed calli express 18 transcription factors also expressed during lateral root initiation (Chupeau et al., 2013). This supports the view that auxin-induced calli have a well-defined gene expression pattern irrespective of the explant and further indicate that callus formation from protoplast-derived cells is not a proof of their dedifferentiation. Rather, senescing leaf cells respond to the artificial hormone treatment with proliferation. Overproliferation of the protoplast-derived cells results in callus formation in the continuous presence of exogenous (or endogenous, in habituated cultures) auxin and cytokinin. Grafi and co-workers (Grafi, 2004; Damri et al., 2009; Grafi et al., 2011a,b; Florentin et al., 2013) recommend considering stress-treated or senescing leaf cells and protoplast-derived cells as dedifferentiated stem cell-like cells since they have (i) open chromatin, (ii) capable to develop in three ways in response to hormonal signals such as elongation, division, or death. This is, however, a very loose interpretation of stem cell-ness and developmental potency.

In conclusion: The term “dedifferentiation” is deeply embedded in the terminology of plant science. In the context of plant biology, it can be defined as a type of transdifferentiation leading to increased developmental potency and/or cell proliferation. Alternatively, the general term “cellular reprogramming” could be used to describe these processes (see, e.g., Ikeuchi et al., 2018). Furthermore, by definition, a “dedifferentiated cell” is a cell that was formed by dedifferentiation. The “dedifferentiated cell state” is a relative developmental term and neither the description of the cell’s genetic landscape nor its developmental potency.

Callus is a result of cellular/tissue reprogramming due to conditions overriding cell/tissue differentiation constrains (hormone gradients, chromatin regulation, cell division block, etc.). Callus can only be considered as a “dedifferentiated” tissue if the above plant-specific definition for dedifferentiation is considered. Callus tissues of various origin can express a wide variety of genes which discriminate them, especially at the early phases of their development. Despite the fact that calli can be formed via various initial pathways, established callus tissues seem to be characterized by a network of transcription factors that facilitate cell fate switch and regeneration. Based on this, the callus is a transient tissue, similarly to the blastema of animals, but can be long maintained under artificial conditions.

## Totipotency and Somatic Embryogenesis

The term “totipotent” has two basically different interpretations: (i) capable of developing into a complete organism or (ii) capable of differentiating into any cell types of an organism (Condic, 2014). In the first and stricter sense, only zygotes or one-celled embryos are totipotent. In the second and wider sense cells which can develop to all the various cell types of an organism but under different condition each, are also totipotent. Based on this second definition, embryonic animal stem cells that can produce a wide range (but not all!) cell types are often considered to be totipotent (Condic, 2014). Since these definitions describe two significantly different developmental potencies, Condic recommended using the term “omnipotent” to suit to the second definition (Condic, 2014).

One can often meet the overstatement, even in university textbooks, that “all/most plant cells are totipotent.” This is based on the erroneous belief that if we can regenerate a whole plant from a cell/explant that evidences cellular totipotency. However, plant regeneration from a totipotent cell must fulfill two main criteria: (i) it must be initiated in an individual cell since totipotency is a cellular term (Condic, 2014); (ii) it must proceed autonomously as a single process (Verdeil et al., 2007).

Whole plants are regenerated from *in vitro* cultured plant cells either directly or indirectly (intervened by callus formation) via organogenesis or somatic embryogenesis. These processes are not autonomous but needs to be induced! Therefore, one could say, at best, that plant cells can (re)gain totipotency but they are not totipotent *per se*. Plant regeneration via several steps obviously does not fulfill the criterium of autonomous development. For example, plant regeneration via organogenesis includes at least two stages: either shoot or root is regenerated from the initial cell and a second induction step is required to regenerate the missing plant part. Not the same cell is forming the shoot and the root! In these processes, the initial cells of root/shoot development can be considered as pluripotent cells. Furthermore, auxin-induced organ regeneration (including callus formation) was shown to initiate in “pericycle-like stem cells” in various tissues and not in somatic cells (Sugimoto et al., 2010). The direct *de novo* formation of stem cells from single differentiated somatic cells is widely believed to take place but hardly evidenced (Gaillochet and Lohmann, 2015; Perez-Garcia and Moreno-Risueno, 2018). Root formation on leaf explants detached from Arabidopsis plants might represent an example (Liu et al., 2014). However, endogenous callus formation initiated with the division of “pericycle-like stem cells” has recently been associated with this regeneration pathway as well (Bustillo-Avendaño et al., 2018). The capability for *de novo* meristem formation is mostly confined to callus tissues (Perez-Garcia and Moreno-Risueno, 2018). During these regeneration processes, appropriate hormonal gradients are established in the callus tissue leading to stem cell niche formation and stem cell differentiation (Perez-Garcia and Moreno-Risueno, 2018). Therefore, the new meristem does not have a clear single cell origin. Moreover, only the newly formed stem cells but not all cells of the callus tissue can be regarded as pluripotent.

Somatic embryogenesis is believed to be the definitive proof for the totipotency of somatic plant cells. Indeed, single cells forming embryos (embryogenic cells) are totipotent by definition since embryos can autonomously develop to whole plants. If all plant cells are totipotent, all plant cells could be able to form somatic embryos. This is obviously not the case. Although somatic embryogenesis is prevalent, it is confined to defined genotypes, developmental states, and explants.

Similarly, to organogenesis, somatic embryogenesis needs induction. This means that although certain somatic cells might (re)gain totipotency under appropriate conditions, they are not totipotent *per se*. Furthermore, somatic embryo formation not necessarily involves neither dedifferentiated somatic nor totipotent cells. Such as callus formation and organogenesis, initiation of embryos from cells surrounding the veins (often referred as procambial cells) was frequently observed (Guzzo et al., 1994; Rose et al., 2010; de Almeida et al., 2012). Whether in these cases embryogenesis shares the initial steps of lateral root/callus formation from “pericycle-like stem cells” still needs to be experimentally addressed. In carrot, somatic embryo formation could be tracked back to single cells or small cell clusters of perivascular origin in the fresh liquid culture of hypocotyl explants (Schmidt et al., 1997). In the presence of auxin (2,4-D), these cells form proembryogenic cell masses (PEMs) as a transitional stage toward embryogenesis. It is a second signal, the removal of auxin, that triggers embryo formation from PEMs (de Vries et al., 1988; Rose et al., 2010). These series of events question the direct autonomous development of somatic embryos from the single embryogenic cells. However, PEMs themselves might be regarded as overproliferating somatic embryos losing their organization (for review, Dudits et al., 1995).

**FIGURE 3:**
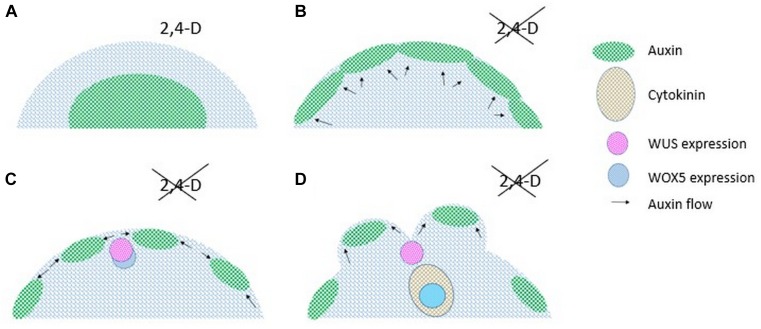
The schematic process of the early steps of multicellular somatic embryo formation on the surface of embryogenic calli. Embryogenic calli formed in the presence of 2,4-dichloro-phenoxyacetic acid (2,4-D) are blocked in development until the removal of this exogenous artificial auxin **(A)**. Following 2,4-D removal, endogenous auxin starts to get produced and start to accumulate at the periphery due to directional auxin transport mediated by the PIN1 auxin efflux carrier proteins **(B)**. Changes in PIN1 orientation result in auxin accumulation in patches at the callus surface. In between, at auxin minima, the expression the WUS transcription factor appears and that partly overlaps with that of WOX5 **(C)**. Cotyledon primordia get organized at auxin maxima and the organizing center of the shoot meristem forms from the cells expressing WUS. The root meristem develops from the region accumulating cytokinin and expressing WOX5 **(D)**. Based on the experiments described in Su et al. (2009, 2015) and Bai et al. (2013). Note that PIN1 is not shown for simplicity.

Recent observations indicate that indirect embryogenesis progresses on surfaces of embryogenic calli via the reorganization of cell clusters instead of developing from single totipotent cells (for review, Su and Zhang, 2014). Several cellular and molecular steps of embryo formation have been revealed in the case of embryogenic Arabidopsis calli (Su et al., 2009, 2015; for review, Fehér et al., 2016). The following model could be established using fluorescent gene expression markers and confocal laser scanning microscopy (Su et al., 2009, 2015; Bai et al., 2013; Figure 3). Embryogenic calli form in the 2,4-D-containing culture medium. Moving them to auxin free conditions, the endogenous synthesis of auxin is induced. The synthesis takes place in the peripheral region of the calli via the expression of YUCCA (YUC) genes (Bai et al., 2013). Subsequently, the synthesis of the PINFORMED1 (PIN1) auxin transport proteins is induced. Their organized orientation results in auxin accumulations in peripheral cell clusters (Su et al., 2009). In between, in the regions exhibiting auxin minima, the gene coding for the WUSCHEL (WUS) meristem identity regulator starts to be expressed (Su et al., 2009). At this early state, the expression of WUS-RELATED HOMEOBOX 5 (WOX5), a master regulator of root meristem organization, partly overlaps with that of WUS (Su et al., 2015). At the next step, cotyledon primordia get organized at the places of auxin maxima on the callus periphery. At this time, cytokinin accumulation can be detected below the WUS expression domain and WOX5 expression is confined to this cytokinin-rich region. The site of WOX5 expression relates with root meristem emergence. In this way, the apical basal axis of the embryo is established before somatic embryos are even visible (Su et al., 2009, 2015; Bai et al., 2013). The above experimental observations indicate the formation (organization) of somatic embryos from groups instead of single callus cells. The induction and reorganization of hormone synthesis and distribution within the callus tissue results in the parallel formation of shoot and root meristems that is followed by the development of an embryo-like structure. This model argues that the regression to a fully dedifferentiated (totipotent) cellular state is not an absolute prerequisite for embryo regeneration from plant tissues.

Nevertheless, the possibility that somatic embryos can form from single somatic cells, cannot be ruled out. Plants has the inherent capability to develop totipotent cells in their soma. During flower formation, plant germ lines develop from well-defined differentiated somatic cells (Shi and Yang, 2010; Twell, 2011). The pathway leading to egg cell formation starts with the differentiation of the archespores in the subepidermal cell layer of the developing ovule and proceeds further with megasporogenesis and embryo sac development (megagametogenesis). Egg cell totipotency is normally suppressed until fertilization (Feng et al., 2013). However, in gametophytic apomixis, diploid egg cells form and can directly develop into zygotes and embryos indicating egg cell totipotency (Koltunow, 2012). It is likely that totipotency of the egg cell is established during the megagametogenesis stage. Egg cell fate is determined by the auxin gradient within the embryo sac (Pagnussat et al., 2009). Cells mispositioned within the embryo sac due to mutations change their fate depending on the auxin concentration (Sundaresan and Alandete-Saez, 2010). Moreover, manipulation of auxin distribution in the embryo sac alters cell fates (Pagnussat et al., 2009). The egg cell forms at a position with high local auxin concentration. Initiation of *in vitro* embryo development from somatic tissues is also associated with high concentration of exogenous and/or endogenous auxin (Fehér et al., 2003). It is tempting to speculate that somatic embryo development initiates with an “egg cell/zygote-like totipotent state” via similar processes taking place in the embryo sac during egg cell differentiation.

However, data providing evidence for the expression of molecular markers of zygotic development during the acquisition of the embryogenic cell fate by somatic cells are missing. Somatic embryogenesis was often reported to start with an asymmetric cell division resembling that of the zygote (Rose et al., 2010). Following the asymmetric division of the Arabidopsis zygote, the WOX2 and WOX8 transcription factors segregate into the apical and basal cells, respectively (Haecker et al., 2004) resulting in the formation of the embryo proper (apically) and the suspensor (basally). These transcription factors (together with WOX9) define the apical-basal developmental pattern of the developing embryo. The above WOX transcription factors have already been implicated in somatic embryo development based on gene expression data (Palovaara and Hakman, 2008; Palovaara et al., 2010; Gambino et al., 2011). However, their expression was not detected yet at the earliest initiation phase. The resemblance of zygotic and somatic embryogenesis is also supported by the development of more-or-less degenerated suspensor-like structures in certain somatic embryogenesis systems (Smertenko and Bozhkov, 2014). The asymmetric divisions of single embryogenic cells can take place even in liquid media indicating that the division asymmetry is defined by intrinsic mechanisms (Dudits et al., 1991). Only the analysis of asymmetrically dividing single cells devoted to the embryogenic pathway could answer the question, how much the first steps of direct somatic and zygotic embryogenesis converge. This kind of approaches are now feasible due to recent advances in the sequencing of single cell transcriptomes.

The polarity of the Arabidopsis zygote is specified by the transcription factors WRKY2 and GROUNDED (GRD)/RKD4 (Ueda and Laux, 2012). Constitutive RKD4 expression caused overproliferation, transient RKD4 expression, however, induced the development of somatic embryos from Arabidopsis root cells (Waki et al., 2011). Transient RKD4 expression likely induced an egg cell/zygote-like cell state in certain root cells. These cells subsequently followed the autonomous embryogenic pathway. AtRKD4 expression was shown to switch on the transcription of those genes in the root that are normally associated with early embryo development. The expression of RKD4 could serve as a tool to validate the zygote-like single cell origin of somatic embryos. Until now, the expression and role of RKD4 or its homologs in non-zygotic embryogenesis have not been demonstrated. Such an approach would require a detection technique with very high sensitivity since RKD4 expression in the zygote is very low (Waki et al., 2011).

Recent transcriptomic comparison indicates that zygotic and somatic embryogenesis can follow rather different pathways and somatic embryogenesis have a gene expression pattern more like germinating seeds (Hofmann et al., 2019). A network of transcription factors [BABY BOOM (BBM), LEAFY COTYLEDON1 (LEC1), LEAFY COTYLEDON2 (LEC2), FUSCA (FUS3), ABSCISIC ACID INSENSITIVE3 (ABI3), AGAMOUS-LIKE15 (AGL15)] governing seed maturation plays central role in many somatic embryogenesis systems (Radoeva and Weijers, 2014; Horstman et al., 2017). The overexpression of these genes can result in ectopic embryo development in vegetative tissues, such as in cotyledons, or at least can promote somatic embryo formation under appropriate conditions. In seedlings, these transcription factors are suppressed not to interfere with vegetative development (Holdsworth et al., 2008). This suppression is mediated by chromatin-remodeling involving among others the PICKLE (PKL) chromatin-remodeling ATPase (Rider et al., 2003; Henderson et al., 2004). Ectopic expression of the above embryo identity factors (e.g., LEC1) in the *pickle* mutant resulted in somatic embryo development (Rider et al., 2003). Mutations in several other chromatin regulators were also shown to allow ectopic embryo formation in vegetative tissues (Tanaka et al., 2008; Tang et al., 2012; Fehér, 2015; Ikeuchi et al., 2015). It is hypothesized, therefore, that embryo development is the default developmental pathway that is suppressed at the chromatin level in vegetative tissues. In this view, embryogenesis cannot be induced but can be released in somatic cells. Unicellular root hair cells were shown to develop into somatic embryos in Arabidopsis mutants not expressing the chromatin regulator POLYCOMB REPRESSIVE COMPLEX 2 (PRC2) (Ikeuchi et al., 2015). This process might involve cellular totipotency since it starts in a single cell. The appearance of somatic embryos, however, was preceded with callus formation. Thus, the multicellular origin of the embryos cannot be excluded either. Even if cellular totipotency is established in chromatin remodeling mutants that is likely different form that of the zygote. The loss of PRC2 function was associated with the ectopic expression of the embryo identity transcription factors (e.g., LEC1, LEC2, FUS3) in the root (Ikeuchi et al., 2015). The expression of these factors could not be detected earlier then the eight-celled stage during zygotic embryogenesis and their earliest roles were observed at the globular stage when the *lec1* mutant exhibits aberrant cell divisions in the suspensor (Harada, 2001). Therefore, somatic embryogenesis in the above transgenic or mutant plants might jump over the first steps of zygotic embryogenesis.

Transient WUS overexpression can also trigger embryo development in vegetative tissues (Zuo et al., 2002). WUS-triggered embryogenesis starts with an asymmetric cell division that may indicate direct embryo formation (Zuo et al., 2002). WUS is a shoot meristem identity factor the expression of which is detected in the zygotic Arabidopsis embryo from the dermatogen stage onward (Mayer et al., 1998). Moreover, WUS was shown to repress LEC1 expression suggesting that WUS cannot activate the embryo identity pathway (Zuo et al., 2002). One can suppose that WUS overexpression can directly induce the formation of shoot meristems (for review, Tian et al., 2018). This might be followed by the reorganization of hormone gradients allowing the subsequent organization of the root meristem. In this way, the apical-basal axis of the forming embryo is established similarly as was observed during indirect somatic embryo formation on embryogenic calli (see earlier). However, the direct reprogramming of certain somatic cells into embryogenic ones by ectopic WUS expression cannot be excluded. This is supported by the experiments where the BABY BOOM (BBM) transcription factor-mediated somatic embryogenesis is enhanced by the co-expression of WUS (Lowe et al., 2016).

The existence of at least two different somatic embryogenesis pathways is supported by the observation made using Arabidopsis immature zygotic embryo explants (Gaj et al., 2005). In this system, the 2,4D-induced direct (callus-free) formation of somatic embryos was found to be LEC1 dependent, but the *lec1* mutants could still form somatic embryos via an indirect WUS-centered pathway.

**FIGURE 4:**
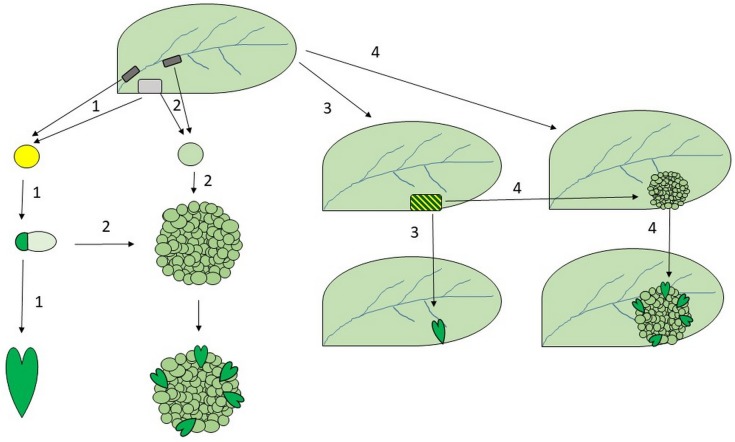
Possible ways of somatic embryo formation. Somatic embryo development can potentially be initiated from differentiated somatic cells (light gray) or from pericycle-like stem cells (dark gray) and can proceed via direct (1,3) or indirect (2,4) pathways *in vitro* (1,2) or *in planta* (3,4). Somatic embryogenesis may start with the induction of single (zygote-like?) totipotent cells (yellow) that form proembryos (dark green) following an asymmetric first division (1). Somatic embryos may be organized on the surface of embryogenic calli from multiple cells (2). *In planta* somatic embryogenesis due to the mutation or ectopic overexpression of regulatory genes can also be direct (3) or indirect (4). If this process starts in a single embryogenic cell (3) than that cell can be considered as totipotent (yellow with dark green lines) but its expression pattern is likely different from that of a hypothetical “zygote-like” totipotent cell (yellow; in processes 1). Note that neither the differentiated somatic cells nor the pericycle-like stem cells are totipotent *per se*, and somatic embryos may form without the participation of single totipotent cells (process 2; see also Figure 3). The drawings are not at scale.

In conclusion: Not all plant cells are totipotent, but under appropriate conditions certain cells may become totipotent. A cell (and only a single cell) can be considered as totipotent if it is able to autonomously develop into a whole plant via embryogenesis. However, somatic embryogenesis is not strictly reliant on cellular totipotency. Theoretically, the development of embryos from somatic cells can initiate in at least three main ways: (1) direct embryogenesis from single cells through a totipotent (zygote-like?) stage; (2) direct or indirect embryogenesis dependent on the embryo-identity transcription factors (LEC1, LEC2, FUS3, etc.); (3) organization of embryos from groups of cells dependent on auxin and cytokinin gradients linked to the parallel establishment of meristem organizing centers (WUS and WOX5 expression) (Figure 4). Therefore, attempts to identify key physiological/molecular/genetic triggers that are valid for all somatic embryogenic systems will obviously lead to failure.

## Author Contributions

The author confirms being the sole contributor of this work and has approved it for publication.

## Conflict of Interest Statement

The author declares that the research was conducted in the absence of any commercial or financial relationships that could be construed as a potential conflict of interest.
